# Protective Effect of Stachydrine Against Cerebral Ischemia-Reperfusion Injury by Reducing Inflammation and Apoptosis Through P65 and JAK2/STAT3 Signaling Pathway

**DOI:** 10.3389/fphar.2020.00064

**Published:** 2020-02-18

**Authors:** Li Li, Lili Sun, Yan Qiu, Wenjun Zhu, Kangyuan Hu, Junqin Mao

**Affiliations:** ^1^Department of Pharmacy, Shanghai Pudong New Area People's Hospital, Shanghai, China; ^2^Department of Pharmacy, Shanghai Punan Hospital, Shanghai, China

**Keywords:** stachydrine, ischemia-reperfusion injury, PC12, oxygen-glucose deprivation, anti-inflammatory

## Abstract

Stachydrine, a constituent of Leonurus japonicus Houtt which also called Japanese motherwort has been shown to improve vascular microcirculation and ameliorate endothelial dysfunction. This study investigated the neuroprotective effect of stachydrine. Male Sprague-Dawley (SD) rats were randomly divided into sham, control, and stachydrine groups. The neurological deficit score was evaluated and the infarct size of the brain was measured using 2,3,5-triphenyltetra-zolium (TTC) chloride staining assay, and the pathological changes in the brain tissues were examined by HE staining. Nissl and terminal deoxynucleotidyl transferase deoxyuridine triphosphate nick end labeling (TUNEL) staining were performed to assess the numbers of Nissl bodies and the levels of apoptosis in the neurons. The activities of superoxide dismutase (SOD) and the levels of malondialdehyde (MDA) were also measured. The release of inflammatory factors IL-1β and TNF-α were detected by Enzyme-linked immunosorbent assay (ELISA). Compared with the control group, the stachydrine group showed a significant prevention of neurological deficit, as indicated by the reduced infarct volume in the brain. Moreover, the stachydrine treatment reduced the activities of SOD, the levels of MDA and decreased the amount of IL-1β, and TNF-α, indicating that it could function to decrease the level of inflammation, thus reducing brain damage. The ischemic stroke model of PC12 cells was prepared *via* oxygen-glucose deprivation (OGD) protocol for 6 h. The expression of P65 and JAK2/STAT3 signaling pathway related proteins was measured by western blot. The treatment group was found to have the survival rate of PC12 cells improved and the release of inflammatory factors reduced when compared with the OGD group. This study demonstrated that stachydrine could improve nerve function by inhibiting the phosphorylation of P65/JAK2 and STAT3.

## Introduction

Stroke is the second leading cause of death in the industrialized countries and the leading cause of acquired adult disability (Go et al., 2013). Focal brain ischemia is usually caused by ischemic stroke, which account for around 80% of all stroke cases. It was estimated that there would be 1.5 million stroke patients in Europe each year till 2025 (Leech et al., 2019; A.K. Boehme et al., 2017). Currently, thrombolysis is well recognized to be the only effective therapy for stroke; however, approximately 5% of the patients treated this way are at a high risk of intracranial hemorrhagic transformation (Campos et al., 2013). Ischemic stroke was reported to show a complex pathophysiological course involving a plethora of distinct molecular and cellular pathways (Wang et al., 2014). Therefore, it is still imperative that we pursue a consistently effective therapy for stroke.

In the past few years, over one hundred traditional Chinese medicine (TCM) patents have been registered for ischemic stroke treatment in China (Chen et al., 2013), among which are the therapies for ischemic reperfusion injury (Moskowitz et al., 2010). Japanese motherwort has been traditionally used to treat some gynecological diseases with blood-circulation problems in East Asia for centuries, the cardio-cerebrovascular benefits of which have been reported from experimental and clinical studies (Zhang et al., 2018). Motherwort has antioxidant properties; leonurine as one of its important constituents has been reported to protect the brain in rats by exerting antioxidant and anti-apoptosis effects (Zhang et al., 2018). It has also been found to improve cerebral ischemia-reperfusion injury in rats (Loh et al., 2010). Stachydrine, a major constituent of motherwort, can exhibit protective effects on vascular endothelial cells (ECs), as indicated by a recent study which reported that stachydrine reversed the Hcy-induced endothelial dysfunction and prevented eNOS uncoupling by increasing the expression of GTPCH1 and dihydrofolate reductase (DHFR) (Servillo et al., 2013; Xie et al., 2018). The low doses of stachydrine could inhibit hydrogen peroxide and induce myometrial smooth muscle cell apoptosis by upregulating Bcl-2 expression (Liu Xin et al., 2012). In the hippocampus, it could improve pathological changes by inhibiting inflammatory reactions after ischemia (Miao et al., 2017). Additionally, stachydrine has been shown to exert anti-oxidant effects by reducing plasma lactate dehydrogenase (LDH) activity in the animal models of acute myocardial ischemia (Liu Xin et al., 2012; Zhao et al., 2017). However, there is a dearth of literature on whether stachydrine can function to treat stroke effectively.

In this study we focused on rats and PC12 cells, because stachydrine was reported to produce a protective effect on cerebral ischemic reperfusion damage in rats, thus enhancing the survival of PC12 cells after oxygen-glucose deprivation (OGD). The OGD cell model was prepared to explore the underlying molecular mechanism of stachydrine's effects; consequently, stachydrine was found to be capable of reducing the cell death rate and improving neuronal function recovery.

## Materials and Methods

### Reagent

**Figure 1:**
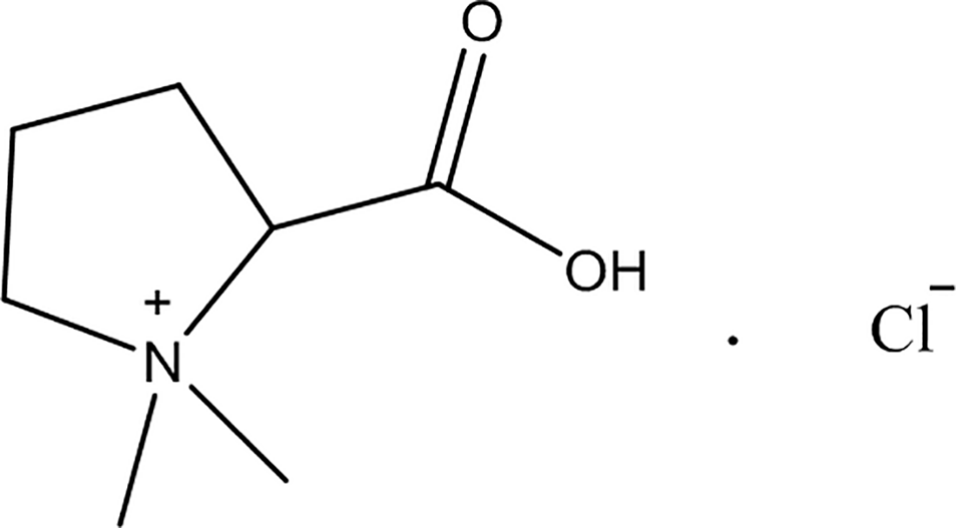
The chemical structure of stachydrine. Stachydrine (purity> 97%) purchased from Dalian Meilun Biology Technology Co. (Dalian, China).

For this study were purchased stachydrine hydrochloric (purity > 97%; Dalian Meilun Biology Technology Company, Dalian, China); 2,3,5-triphenyltetrazolium chloride (Sigma, MO, USA); total RNA Kit (Takara, Shiga, Japan); ELISA Kit (R&D Systems, MN, USA); the antibodies of P65 (1:1,000), p-P65 (1:1,000), ikB (1:1,000), p-ikB (1:1,000), JAK2 (1:500), p-JAK2 (1:1,000), STAT3 (1:1,000), and p-STAT3 (1:1,000; Cell Signaling Technology Danvers, MA, USA); and goat anti-rabbit immunoglobulin G (IgG) (Cell Signaling Technology, Danvers, MA, USA). As illustrated in **Figure 1**, the structure of stachydrine was presented.

### Animal Care and Experimental Protocol

Approved by Ethics Committee of Shanghai Pudong New Area People's Hospital, this study was carried out in accordance with the Basel Declaration's recommendations of the care and use of laboratory animals. Purchased from Shanghai Experimental Animal Center of Chinese Academy of Sciences (Shanghai, China), the rats were housed in the controlled environment under a 12 h light/dark cycle, and fed with standard rat chow and water. The male Sprague-Dawley rats weighing 260–280 g were housed in diurnal lighting condition, with free access to food and water. All rats were divided randomly into sham-operation group; control group, i.e., Middle Cerebral Artery Occlusion (MCAO) group injected with saline solution; and stachydrine groups, i.e., MCAO group injected once with stachydrine hydrochloric (Sta 27.93, Sta 55.87, Sta 111.73, Sta 167.60 mM) through the caudal vein 15 min after MCAO surgery. The neurobehavioral deficits were assessed and the brain of each rat was harvested 24 h after MCAO surgery. Those which received stachydrine 167.60 mM were treated, once daily (24 h), with stachydrine hydrochloric for 48 or 72 h through the caudal vein, while the sham and control groups received the same volume of normal saline.

### Cell Culture

Obtained from the Cell Bank of Chinese Academy of Sciences (Shanghai, China), PC12 cells were cultured in Dulbecco's modified Eagle's medium (DMEM) (Hyclone, Logan, UT, USA) and 100 ug/ml penicillin-streptomycin solution (Thermo Scientific, Waltham, MA, USA), which were supplemented with 10% fetal bovine serum (Gibco, Carlsbad, CA, USA). The cells were cultured under the conditions of 37°C, 5% CO_2_, and 95% humidified atmosphere.

### Middle Cerebral Artery Occlusion Surgery

The male adult SD rats were anesthetized with 2% sodium pentobarbital (200 mM,i.p.), the neck shaved and cleaned with 75% ethanol. The left MCAO surgery was performed as previously described (Zhang et al., 2012). The left common carotid artery (CCA) was exposed through an incision in the middle of neck. The external carotid artery (ECA) was tied to prevent bleeding. A 4-0 poly-L-lysine filament (250–300 g) with a blunt end (Beijing Cinontech Co. Ltd, China) was inserted into the CCA to be advanced into the middle cerebral artery *via* the internal carotid artery (ICA) until a mild resistance was felt and the dark mark on the filament was in the vascular bifurcation position (18–20 mm), and then the blood of the middle cerebral artery was occluded. Two hours after the ischemia, the filament was slowly withdrawn until the dark mark was seen. Afterwards, the animals were returned to their cages to allow 24 h-reperfusion. Their body temperature was maintained at 37 ± 0.5°C with a heating lamp during the surgery. In the sham group, ECA was surgically prepared for filament insertion, but it was not inserted. After this surgery, the animals were sent to their cages to recover from anesthesia.

### Neurological Deficit

After MCAO, neural dysfunction was evaluated based on the deficit grading system and according to the classic method introduced by Longa EZ and coworkers (Longa et al., 1989). A scale of 0 to 5 was used to assess the behavioral and motor changes in rats after MCAO surgery. When suspended by the tail, the rats extended both forelimbs toward on the floor, which represented a normal behavior corresponding to a score of 0. The rats were assigned a score of 1 when the contralateral forelimbs were on the side, without other abnormalities observed. The rats were placed on the ground to be allowed to move freely, their circling behavior observed. A score of 2 was assigned to those which moved spontaneously in all directions, but showed a monodirectional circling; a score of 3, to those which showed a consistent spontaneous contralateral circling (Loh et al., 2009); and a score of 4, to those which were very weak and collapsed.

### Infarct Volume of Brain

To the infarct volume of brain was applied TTC staining (Sigma, MO, USA) for assessment. The brain specimens were harvested to be frozen at –20°C for 30 min, and the cerebellum was removed. The brain was sectioned into six pieces of 2 mm thick coronal slices using a new scalpel blade. The sections were stained with 1% TTC solution at 37°C for 20 min before preserved in 4% formaldehyde at 4°C for at least 24 h. The brain infarct areas were analyzed with Image-Pro Plus 6.0 to estimate the infarct volume in the whole hemisphere.

### Hematoxylin and Eosin Staining

When the animals were euthanized, the brain was carefully kept in 4% paraformaldehyde for 24 h. The slices were dyed with hematoxylin and then stained with eosin. The color changes were observed under a microscope to control the degree of dyeing. After dyed, those slices were washed with distilled water and dehydrated with gradient alcohol. Dried at room temperature, the histological changes were examined under an optical microscope.

### Nissl Staining

The paraffinized brain samples were treated with Nissl staining solution (Sangon Biotech, Shanghai, China). Upon dyeing, a bluish-purple color was observed, which displayed the basic nervous structure of the brain. Large and numerous Nissl bodies were observed, indicating that the nerve cells had a high ability to synthesize proteins. When the nerve cells were damaged, however, the number of Nissl corpuscles decreased significantly. The number of stained cells was counted from the randomly selected fields and analyzed with Image-Pro Plus 6.0.

### Terminal Deoxynucleotidyl Transferase Deoxyuridine Triphosphate Nick End Labeling Assay

The brain cells were treated with terminal deoxynucleotidyl transferase deoxyuridine triphosphate nick end labeling (Tunel) assay to determine the number of apoptotic cells. (In Situ Cell Death Detection Kit, Roche, Basle, Switzerland). The slides were heated to 60°C before washed with xylene and rehydrated through a series of concentration gradients of ethanol. The tissue sections were incubated in a working solution for about 20 min before put into the reaction mixture solution. The phosphate buffered saline (PBS) was used to wash the slides for analysis under a microscope. The apoptotic cells were those which had a brown-stained nucleus. The number of apoptotic cells was counted from the randomly selected fields.

### Immunohistochemistry and Immunofluorescence

The brain slices were pretreated with 0.3% H_2_O_2_ and blocked in 0.1% bovine serum albumin for 30 min, before incubated overnight at 4°C with primary antibody P65. After rinsing, they were incubated with goat anti-rabbit IgG secondary antibody for 1 h at room temperature. After that, they were observed under a microscope. Methods double label immunofluorescence were observed with confocal laser microscope.

### Oxygen-Glucose Deprivation

PC12 cells were subjected to the OGD procedure to mimic ischemic conditions *in vitro* as previously described (Zhu et al., 2016). Before OGD, the cells were washed thrice with PBS, and then pretreated with stachydrine and glucose-free DMEM for 1 h. After that, they were incubated in a humidified modular hypoxia chamber (Billups-Rothenberg, Del Mar, USA) with 5% CO_2_ and 95% N_2_ for 6 h. The chamber was placed in an incubator set at 37°C. A normal normoxia medium served as the control.

### Real-Time Polymerase Chain Reaction Assay

Total RNA was isolated from the cells subjected to OGD using Total RNA Kit (Takara, Shiga, Japan), and the complementary DNAs (cDNAs) were synthesized by 5×Primescript reverse transcription reagents (Takara, Shiga, Japan) following the manufacturer's instructions. Real-time (RT)-PCR was performed using SYBR Premix ExTaq™ (Tili RnaseH Plus; Takara, Shiga, Japan) on 7500 Real-Time PCR System (Applied Biosystems). The primers were used as follows:

Rat TNF-α ForwardAAATGGGCTCCCTCTCATCAGTTC
ReverseTCTGCTTGGTGGTTTGCTACGAC
Rat IL-1βForwardAGGCTGACAGACCCCAAAAG
ReverseCTCCACGGGCAAGACATAGG
Rat GAPDHForwardACCACAGTCCATGCCATCAC
ReverseTCCACCACCCTGTTGCTGTA

### Enzyme-Linked Immunosorbent Assay

ELISA was employed to determine the release of inflammatory cytokines IL-1β and TNF-α in serum and the supernatant of the rats and PC12 cells. The examination of IL-1β and TNF-α was carried out according to the instructions of the kit (R&D Systems, MN, USA).

### Western Blot Analysis

PC12 cells were kept at a density of 1.5×10^5^ cells/ml in six-well plates for 24 h. After OGD, the protein was isolated from the whole cell lysate with M-PER Protein Extraction Reagent (Pierce, Rockford, IL) supplemented with protease inhibitor cocktail. The protein was resolved on sodium dodecyl sulfate polyacrylamide gel electrophoresis (SDS-PAGE) according to the molecular weight, and then transferred to the membranes, which were blocked for 2 h with 5% BSA before incubated with rabbit anti-P65 antibody (1:1,000), p-P65 (1:1,000), ikB (1:1,000), p-ikB (1:1,000), JAK2 (1:500), p-JAK2 (1:1,000), STAT3 (1:1,000), and p-STAT3 (1:1,000) overnight at 4°C. The immunoreactive proteins were detected using enhanced chemiluminescence (ECL) reagents western blotting substrate (Thermo Scientific, Waltham, MA, USA).

### Statistical Analysis

All data was analyzed using SPSS19.0 software and expressed as the mean ± SEM. The significant differences between the groups were examined by one-way analysis of variance (ANOVA). *P* < 0.05 was considered to be statistically significant.

## Results

### The Neuroprotective Effect of Stachydrine on the Neurological Deficit and Infarct Volume

**Figure 2:**
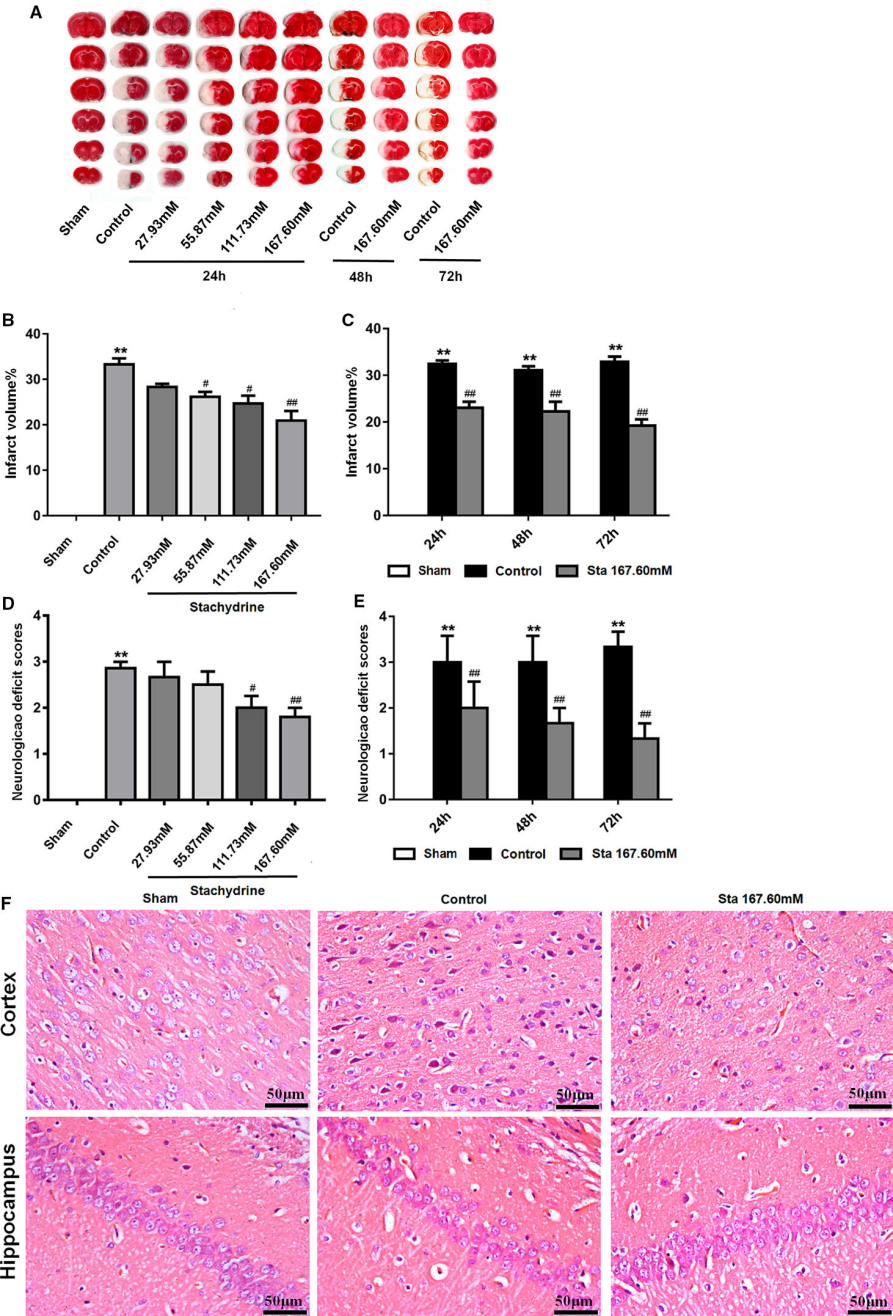
The neuroprotective of stachydrine on neurological deficit and infarct volume. The neuroprotective effect of stachydrine on the neurological deficit and infarct volume of the brain was stained by tetrazolium chloride (TTC) and HE. **(A)** TTC staining used to assess the infarct areas of the brain. **(B)** The brain infarct areas analyzed with Image-Pro Plus 6.0 to estimate the infarct areas in the whole hemisphere after MCAO; **P < 0.01, Sham group vs. Ccontrol group, ^#^*P* < 0.05, ^##^*P* < 0.01 *vs.* control group, n = 6. **(C)** The infarct areas in the whole hemisphere of stachydrine (167.60 mM) treatment after MCAO 24, 48, and 72 h, analyzed; **P < 0.01, Sham group vs. Ccontrol group, ^##^*P* < 0.01 *vs.* control group, n = 6. **(D)** Effect of stachydrine on the neurological scores after MCAO; **P < 0.01, Sham group vs. Ccontrol group, ^#^*P* < 0.05, ^##^*P* < 0.01 *vs.* control group, n = 6. **(E)** Effect of stachydrine (167.60 mM) on neurological scores after MCAO 24, 48, and 72 h, detected; **P < 0.01, Sham group vs. Ccontrol group, ^##^*P* < 0.01 *vs.* control group; n = 6. **(F)** Pathological changes in cortex and hippocampus of brain evidenced by HE staining.

The classic method introduced by Longa EZ was used to assess the behavioral and motor changes in the rats after MCAO surgery. The sham group did not show any neurological deficit, but had a neurological score of 0. The control group presented the highest score after the surgery (Longa et al., 1989). The stachydrine group had the scores decrease significantly when compared with the control group. The scores of the control group increased significantly after reperfusion when compared with those of the sham group (2.50 ± 0.25 *vs.* 0.00 ± 0.00; *P* < 0.01). After 24 h-reperfusion, the administration of stachydrine significantly decreased the neurological deficit scores in the stachydrine group when compared with those in the control group (1.50 ± 0.23 [Sta 167.60 mM] *vs.* 2.50 ± 0.25; *P* < 0.01; **Figure 2D**). Moreover, the neurological deficit scores decreased over time (**Figure 2E**).

Since the sham group presented no neuronal injury, no infarct area was observed after TTC stained. Compared with that in the sham group, the cerebral infarct volume increased significantly in the control group. In contrast, the infarct area was significantly reduced in the stachydrine group when compared with that in the control group [22.52 ± 2.5% (Sta 167.60 mM for 24 h) *vs.* 38.99 ± 1.54%; *P* < 0.01; **Figures 2A, B**]. The infarcted area decreased over time (**Figure 2C**).

As indicated by the results of the histopathological changes examined in all groups by HE staining, the brain tissue structure was normal in the sham group. The nerve cells showed a clear outline with rounded nuclei and clear, visible nucleoli. In the hippocampus, the cells appeared orderly. The cerebral tissue in the control group showed edema on the half side of the ischemia-reperfusion injury. The HE staining revealed the presence of many vacuoles in different sizes in the brain tissue, the cells loosely arranged. The degree of cytoplasmic staining was uneven, while the cell nucleus was wrinkled and deformed.

The histopathological changes in the brain tissues were detected in the stachydrine group in comparison to the control group. The neuronal structure was found to be relatively regular, the nuclei visible and the vacuolization smaller without obvious damages both in cortex and hippocampus (**Figure 2F**).

### Stachydrine Ameliorates Neurons Apoptosis

From Nissl staining used to examine the ischemia-reperfusion induced injury of neurons in the cerebral cortex, the results showed that the sham group had bluish-purple color Nissl bodies with orderly nuclei. Moreover, there were many and large neurons with normal structure. In the cortex of the control group, the cells were disorderly, the number of Nissl bodies significantly reduced, the cell gap increased and many vacuoles formed. The stachydrine group increased the number of Nissl bodies, thus leading to a clear nucleus when compared with the control group.

**Figure 3:**
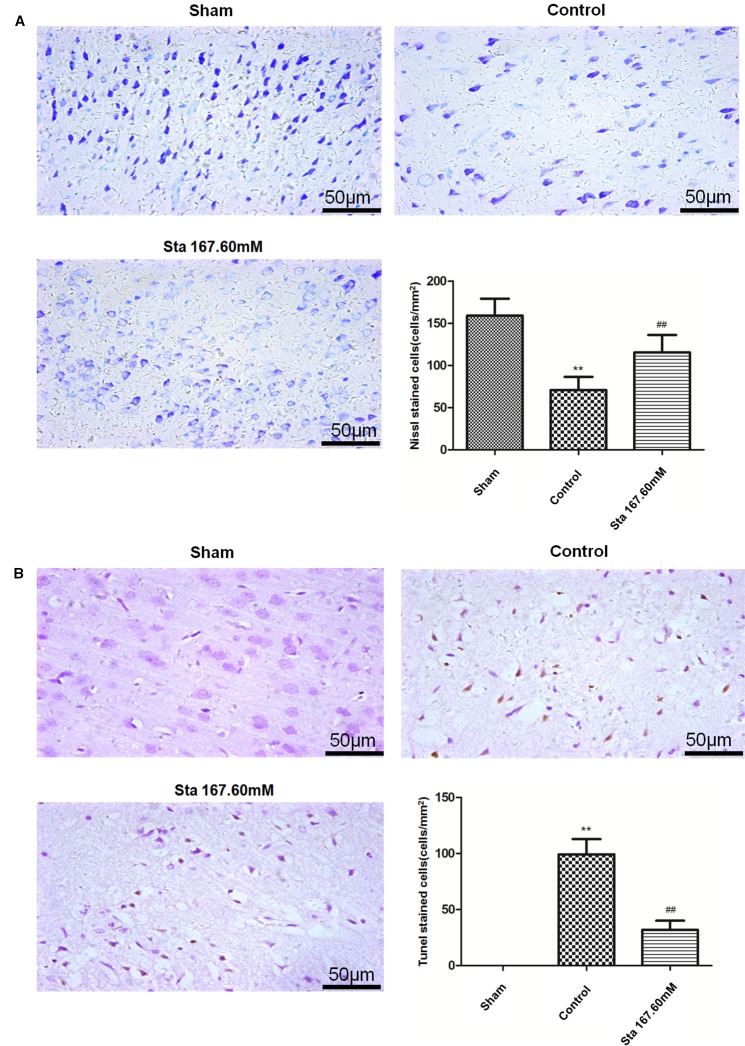
Stachydrine ameliorates neurons apoptosis. Nissl staining and terminal deoxynucleotidyl transferase deoxyuridine triphosphate nick end labeling (TUNEL) staining used to examine the ischemia-reperfusion injury of neurons in cerebral cortex. **(A)** Pathological changes detected by Nissl staining, and the number of Nissl bodies counted; **P < 0.01, sham group *vs.* control group; ^##^*P* < 0.01, stachydrine (167.60 mM) group *vs.* control group, n = 4. **(B)** TUNEL staining of apoptosis in brain and quantification of TUNEL-positive cells, examined; **P < 0.01, sham group *vs.* control group, ^##^*P* < 0.01, stachydrine (167.60 mM) group *vs.* control group, n = 3.

After Nissl staining, the cell number was 159 ± 8.18 cells/mm^2^ in the sham group, but 70.83 ± 76.34 cells/mm^2^ in the control group. The cell number was 115.3 ± 8.47 cells/mm^2^ in the stachydrine group of 167.60 mM, which was significantly higher than that in the control group (*P* < 0.01; **Figure 3A**). Brown karyon staining cells were observed to be apoptotic under Olympus light microscope, which were counted in the groups. Additionally, the treatment of stachydrine decreased the number of apoptotic cells as compared with the control group (*P* < 0.01; **Figure 3B**).

### Decreased Expression of P65 and p-STAT3 in Rat Brain

**Figure 4:**
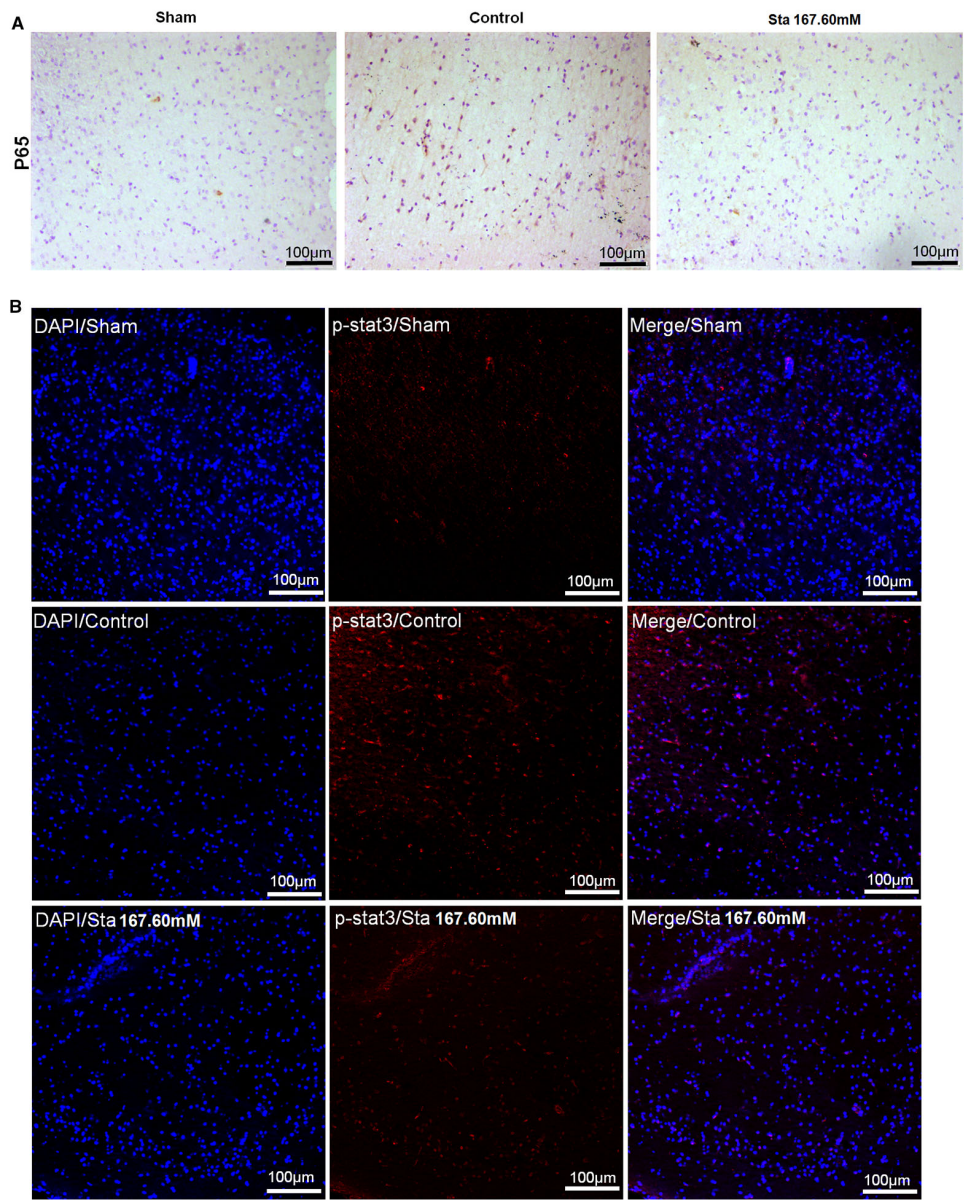
Effect of stachydrine on P65 and p-STAT3 expression. **(A)** Immunohistochemistry staining used to analyze the expression of P65 protein levels after MCAO surgery in rat brain. **(B)** Immunofluorescence staining employed to observe the effect of p-STAT3 protein after ischemia reperfusion injury under confocal microscopy; stachydrine: 167.60 mM.

As revealed by immunohistochemistry staining assay, a decrease was observed in the rat brain section P65 protein levels after MCAO surgery (**Figure 4A**). According to the immunofluorescence staining to determine the expression of p-STAT3 protein in the rat brain section after ischemia reperfusion injury, the treatment of stachydrine decreased the protein expression of p-STAT3 (**Figure 4B**).

### Superoxide Dismutase Activities Increased While Malondialdehyde, IL-1β, and TNF-α Levels Decreased

**Figure 5:**
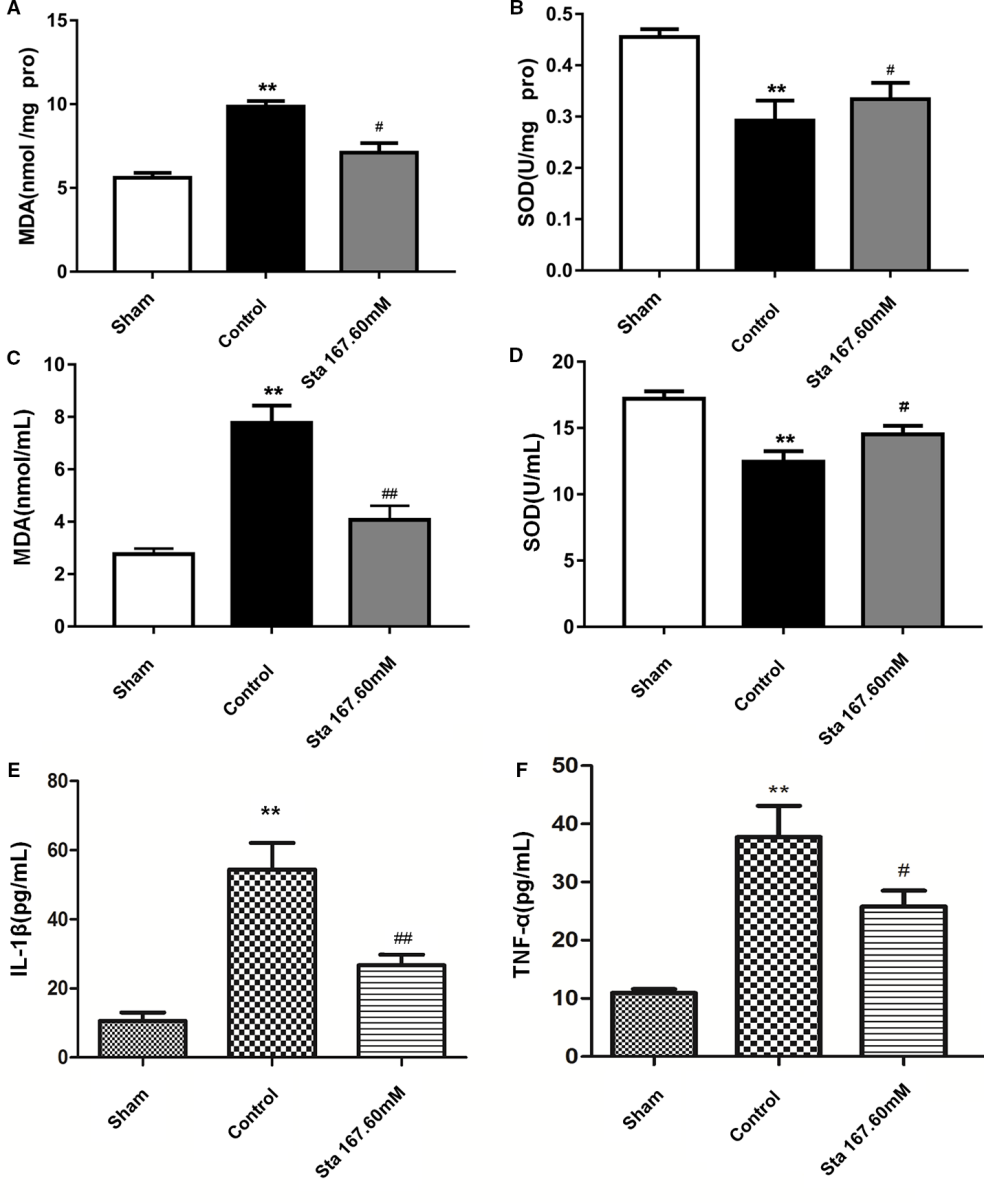
Effect of stachydrine on the activities of superoxide dismutase (SOD) and the levels of malondialdehyde (MDA), interleukin (IL)-1β, and TNF-α MDA and SOD detection kits used to investigate the effect of stachydrine on the levels of malondialdehyde (MDA) and the activities of superoxide dismutase (SOD); ELISA used to detect the IL-1β and TNF-α in serum collected at the time point of 24 h after reperfusion; effect of brain tissue **(A)** MDA, **(B)** SOD, effect of **(C)** MDA, **(D)** SOD, **(E)** IL-1β, and **(F)** TNF-α in rat serum with stachydrine on cerebral ischemia reperfusion injury, examined; stachydrine: 167.60 mM; **P < 0.01, sham group *vs.* control group, ^#^*P* < 0.05, ^##^*P* < 0.01, stachydrine group *vs.* control group, n = 3.

Since oxidative stress plays an important role in ischemia-reperfusion injury, we investigated the effect of stachydrine on superoxide dismutase (SOD) activities and malondialdehyde (MDA) levels in the brain tissue and serum. The stachydrine group showed a decrease in the levels of MDA (*P* < 0.05; **Figure 5A**) as well as in the serum levels of MDA (*P* < 0.01, **Figure 5C**), when compared with the control group, respectively. Moreover, SOD activities were significantly lower in the control group than in the stachydrine group (*P* < 0.05; **Figure 5B**). The serum activities of SOD showed a similar trend (*P* < 0.05; **Figure 5D**).

To examine whether stachydrine treatment could induce an anti-inflammatory pattern, ELISA was employed to detect the levels of IL-1β and TNF-α in serum after 24 h-reperfusion. The sham group had significantly lower levels of IL-1β and TNF-α than the control group (*P* < 0.01). The stachydrine group showed a reduction in the levels of IL-1β when compared with the control group (*P* < 0.01; **Figure 5E**). TNF-α expression was decreased after treatment with stachydrine compared with control group (*P* < 0.05; **Figure 5F**).

### Viability Increased and IL-1β and TNF-α Levels Decreased in PC12 Cells After Oxygen-Glucose Deprivation

**Figure 6:**
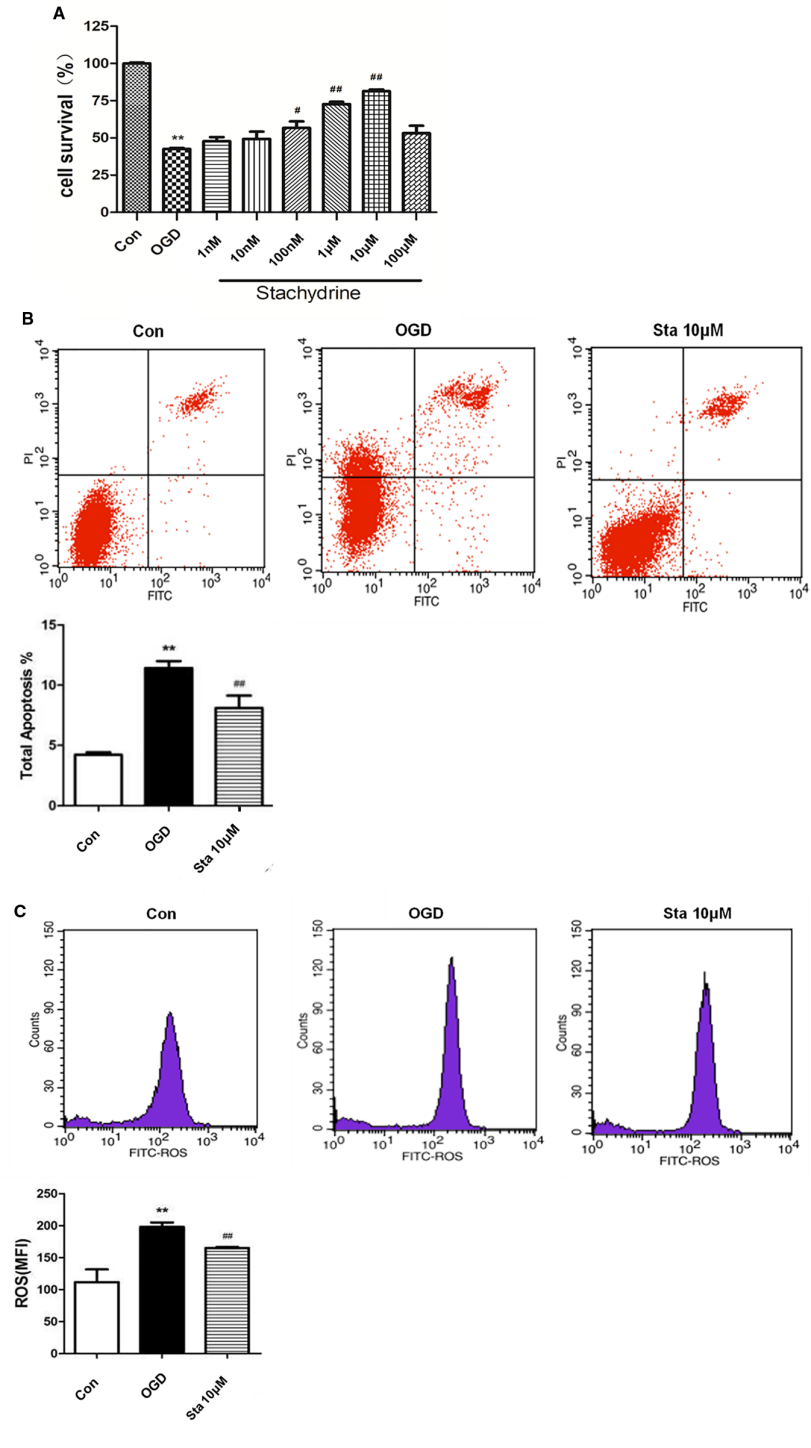
Cell viability in PC12 cells after oxygen-glucose deprivation (OGD). **(A)** MTT used to test PC12 cells viability. **(B)** The flow cytometry results used to show the percentage of total apoptotic cells, as compared with OGD groups. **(C)** The flow cytometry results used to show the level of ROS in PC12 cells; stachydrine: 10 μM; **P < 0.01, control group *vs.* OGD group, ^#^*P* < 0.05, ^##^*P* < 0.01, stachydrine group *vs.* OGD group, n = 3.

When the cell viability was tested by MTT, it was found that the stachydrine group showed an increase in the survival rate of PC12 cells when compared with OGD group (**Figure 6A**). The results of the flow cytometry showed the OGD cells treated with 10 μM stachydrine had a higher percentage of total apoptotic cells than the OGD group (*P* < 0.01; **Figure 6B**). Moreover, ROS was reduced in PC12 cells treated with 10 μM stachydrine, as revealed by the flow cytometry (*P* < 0.01; **Figure 6C**).

**Figure 7:**
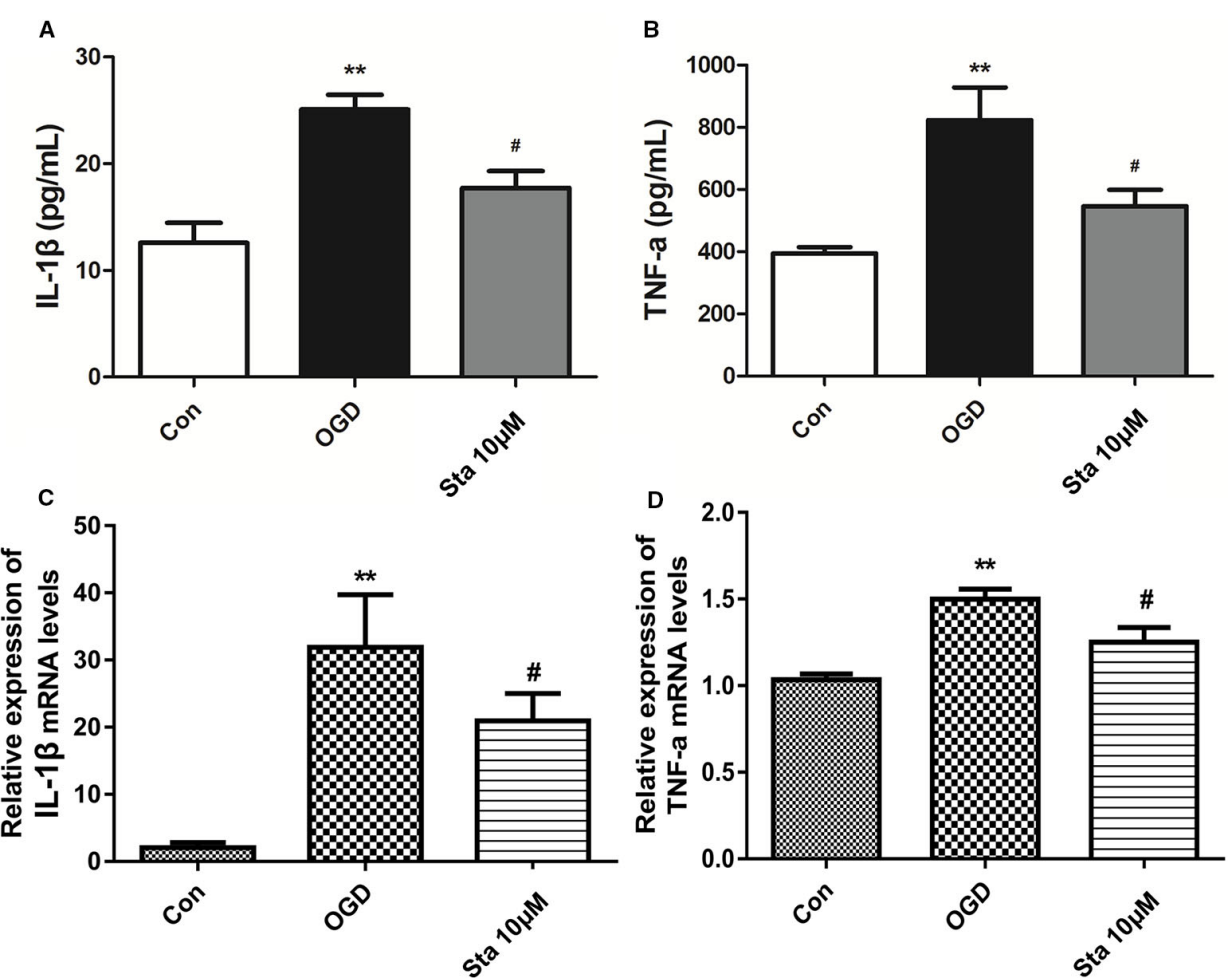
Effect of stachydrine on interleukin (IL)-1β and TNF-α levels in PC12 cells after oxygen-glucose deprivation (OGD). The level of IL-1β and TNF-α in supernatants of PC12 cells measured by ELISA. **(A)** The IL-1β and **(B)** TNF-α levels in supernatants from PC12 cells, examined. Real-time PCR used to evaluate the **(C)** IL-1β and **(D)** TNF-α mRNA levels in PC12 cells; stachydrine: 10 μM, **P < 0.01, control group *vs.* OGD group, *^#^P* < 0.05, stachydrine group *vs.* OGD group, n = 3.

As indicated by the measurement of IL-1β and TNF-α levels in the supernatants of PC12 cells by ELISA, a decrease was observed in IL-1β levels of OGD cells treated with 10 μM stachydrine (*P* < 0.05; **Figure 7A**), and also in TNF-α levels (*P* < 0.05; **Figure 7B**). Real-time PCR assay showed that IL-1β and TNF-α messenger RNA (mRNA) levels were up-regulated after OGD, which, however, were down-regulated after the treatment of stachydrine (*P* < 0.05; **Figures 7C, D**).

### P65 and JAK2/STAT3 Signaling Pathway Suppressed in PC12 Cells After Oxygen-Glucose Deprivation

**Figure 8:**
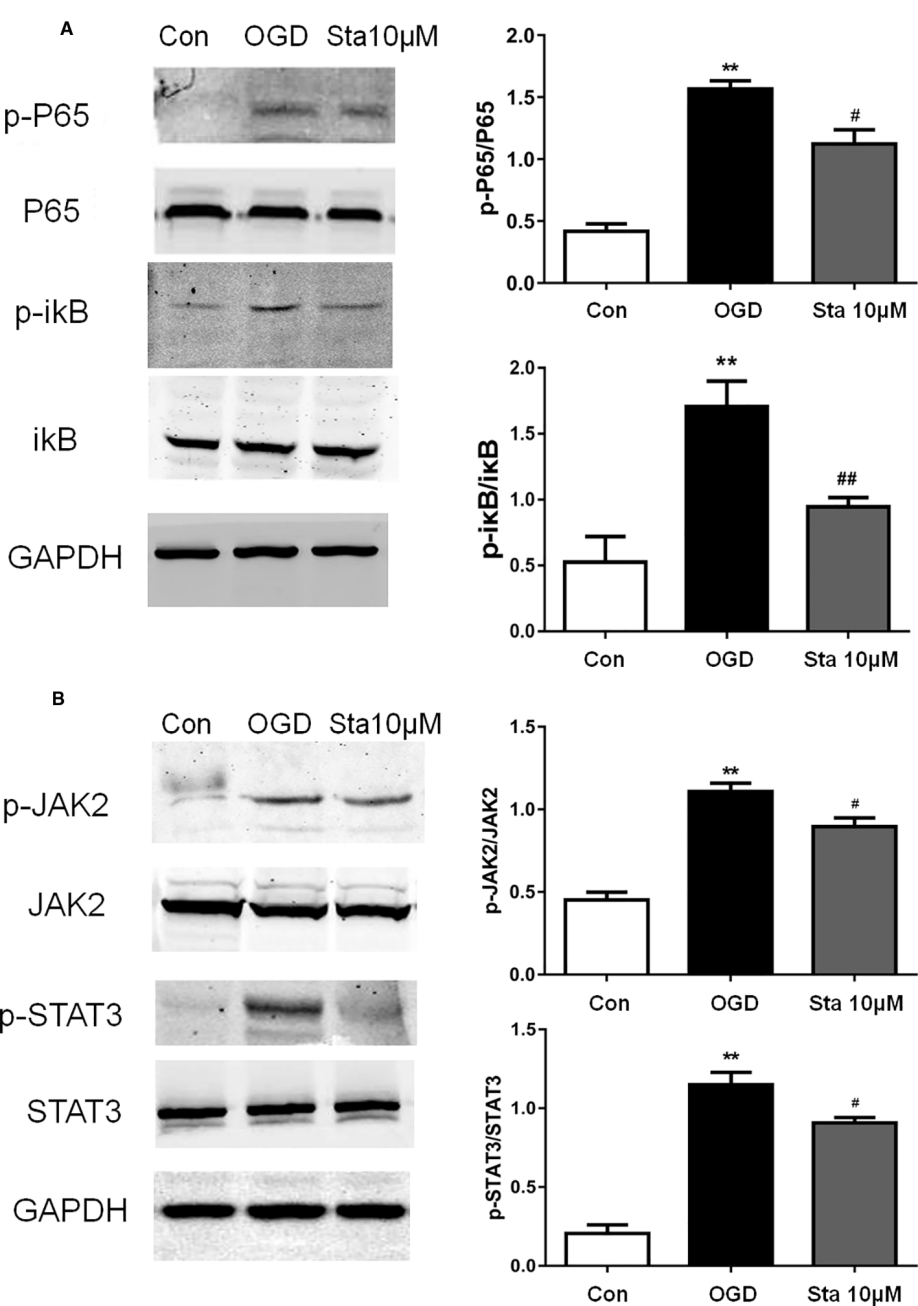
Effect of stachydrine on P65 and JAK2/STAT3 signaling pathway in PC12 cells after oxygen-glucose deprivation (OGD). Western blot used to detect the protein levels in PC12 cells after OGD. **(A)** The expression of p-P65 and p-ikB levels in PC12 cells after oxygen and glucose deprivation, examined. **(B)** The expression of p-STAT3 and p-JAK2 levels in the OGD group, control group, and stachydrine group, examined; stachydrine: 10 μM; **P < 0.01, control group *vs.* OGD group, ^#^*P* < 0.05, ^#^*^#^P <* 0.01, stachydrine group *vs.* OGD group, n = 3.

Since stachydrine was found to have an effect on anti-inflammatory factors of IL-1β and TNF-α, we postulated that it could regulate P65. Indeed, our results showed that the phosphorylation of P65 and p-ikB were decreased in the stachydrine group in comparison with the OGD group (*P* < 0.05; **Figure 8A**). The expressions of p-STAT3 and p-JAK2 were increased in the OGD group in comparison with the control group, while stachydrine pretreatment suppressed the expressions of p-STAT3 and p-JAK2 proteins, as compared with the OGD group (*P* < 0.05; **Figure 8B**).

## Discussion

Over the years, a quite number of drugs have been used in treating stroke, some of them proved to be effectively neuroprotective. However, the currently used drugs are not satisfactory enough to control stroke. In this study, we tested whether stachydrine could inhibit excessive inflammation and oxidative stress *in vitro* and *in vivo*. The *in vivo* results showed that stachydrine improved the pathological changes in the hippocampus, thus preventing neuronal injury, which was similar to the previously reported finding (Miao et al., 2017). Although this finding is in agreement with our results in some ways, but the animal models were different. Another previous study demonstrated that stachydrine suppressed traumatic brain injury *via* anti-inflammatory mechanisms (Yu et al., 2018). The effects of stachydrine on immunity and inflammation have also been reported in recent studies (Cao et al., 2017; Meng et al., 2019).

In the present study, the infarcted areas were identified by TTC staining, and the neurological deficits scores were evaluated after 24 h-reperfusion injury. The treatment of 167.60 mM stachydrine significantly reduced the infarct volume and alleviated the neurological impairment, thereby resulting in lesser histological damage, as compared with the control group.

In view of which, we investigated the underlying therapeutic mechanism of stachydrine. It is well known that necrosis or apoptosis can aggravate ischemic damage. Ischemia-reperfusion injury is associated with inflammatory response and apoptosis, but the exact mechanism is unclear (Mestriner et al., 2013; Fluri et al., 2015; Hu et al., 2015). In the current study, the treatment of stachydrine significantly reduced the number of Tunel-positive cells after ischemia. Nissl staining suggested that the treatment alleviated neuronal injury and significantly increased the number of Nissl bodies. Although oxidative stress is characterized by imbalance between antioxidant defense mechanisms and free radicals, the mechanism of this imbalance is not clear in ischemic-induced apoptosis.

Under normal physiological conditions, the level of ROS is low in cells, and controlled by internal antioxidants without causing damage (Heiler et al., 2011; Wei et al., 2015; Momosaki et al., 2016; Andrienko et al., 2017). Since SOD and MDA are among the key biomarkers of oxidative stress, their levels directly reflect the speed and degree of lipid peroxidation, and indirectly show the level of free radical scavengers (Krupinski et al., 2003; Yan et al., 2017). The reduced activity of SOD and enhanced production of MDA in brain tissue and serum of the control group implied that cerebral ischemia induced severe oxidative stress. In the current study, the effect of cerebral ischemia on the level of these oxidative biomarkers were reversed following the treatment of stachydrine. It therefore followed that the *in vivo* therapeutic effect of stachydrine could be strongly related to the antioxidant effect. When compared with the OGD group, the stachydrine group showed a reduction in ROS levels of PC12 cells, as revealed by flow cytometry.

As one of the key factors involved in stroke development, tumor necrosis factor alpha (TNF-α) can interfere with the normal function of the brain, affecting the permeability of the blood-brain barrier, and impairing the transmission of glutamic acid as well as the plasticity of the synapse. Other studies have reported that TNF-α increased the density of the receptors associated with neurotoxicity (Stephenson et al., 2000; Gu et al., 2013). TNF-α could also be involved in the activation of multiple signaling pathways, such as P65 pathway and JAK2/STAT3 signaling pathway (Qi et al., 2012). IL-1β regulating inflammatory responses as one of the cytokines involved in stroke development, the overexpression of IL-1β could affect the functions of cognition and memory (Zhong et al., 2003). The current results showed that serum of TNF-α and IL-1β was decreased to varying degrees, indicating that the protective effect of stachydrine may be related to the suppression of overall inflammatory response.

JAK/STAT signaling was reported to play a key role in cerebral ischemia-reperfusion injury, remodeling ischemia reperfusion-induced brain dysfunction (Chang et al., 2014). JAK is a tyrosine kinase composed of four family members, JAK1, JAK2, JAK3, and JAK4; JAK1 and JAK2 are widely distributed in various tissues and cells (Kuang et al., 2014; Zhao et al., 2017). STAT could directly transmit signals into the nucleus (Alhadidi and Shah, 2017). STAT3 could regulate the expression of genes encoding proteins involved in inflammation (Murase et al., 2012). Some studies have shown that these proteins participate in disease mechanisms such as inflammation in neurodegenerative disorders, that the JAK2/STAT3 signaling pathway is activated after cerebral ischemia, which can increase the expression of HMGB1 and aggravate post-ischemic inflammatory responses (Saydmohammed et al., 2010; Wu et al., 2018; Zhou et al., 2019). The P65 pathway has been found to mediate the process of ischemic brain injury, hence a promising therapeutic target for ischemic stroke, and ikB as the master inhibitor of the P65 pathway, to have a degrading function during pathway activation (Hayden et al., 2008; Tu et al., 2014; Wang et al., 2017). A recent study has shown that linagliptin could suppress the expression of phosphorylated JAK2, phosphorylated STAT3 and P65 to confer neuroprotection (Marotta et al., 2011; Chang et al., 2018; Elbaz Eman et al., 2018). These results are similar to those which were found in the current study, which suggests the effects of inflammation reduction and neuroprotection.

In the current study, the treatment of stachydrine inhibited the P65 and JAK2/STAT3 signaling *in vitro*, as evidenced by the down-regulation of p-P65, p-ikB, p-JAK2, and p-STAT3, which suggested that stachydrine could protect against OGD injury in PC12 cells.

In conclusion, the current study demonstrated that stachydrine reduced neurological dysfunction, neuronal injury and cerebral infarction in a rat model, which may be associated with the down-regulation of inflammatory processes. This indicates that the treatment of stachydrine could be beneficial to stroke patients, as it prevents reperfusion-induced injury due to its endogenous antioxidant capacity and anti-inflammatory effect.

The current study still had certain limitations. It was to study the protective effect of ischemia-reperfusion injury; thrombolysis was not involved as the main treatment; and stachydrine was mainly administered as an auxiliary treatment as part of the rehabilitation after thrombolysis. Further studies are advocated to design therapeutic agents based on stachydrine to control cerebral ischemia-reperfusion injury.

## Data Availability Statement

All datasets generated for this study are included in the article/**Supplementary Material**.

## Ethics Statement

This study was carried out in accordance with the principles of the Basel Declaration and recommendations of the care and use of laboratory animals, Ethics Committee of Shanghai Pudong New Area People's Hospital. The protocol was approved by the Ethics Committee of Shanghai Pudong New Area People's Hospital.

## Author Contributions

JM conceived the study. LL and KH constructed the animal model. LS and WZ performed the cell experiments, LL analyzed the data and wrote the manuscript. JM and YQ revised the manuscript.

## Funding

This work was supported by the Project of Shanghai University of Medicine & Health Sciences Cooperative Innovation Project (Grant No. SPCI-18-13-001); Important Weak Subject Construction Project of Pudong Health and Family Planning Commission of Shanghai (Grant No. PWZbr2017-16).

## Conflict of Interest

The authors declare that the research was conducted in the absence of any commercial or financial relationships that could be construed as a potential conflict of interest.

## Supplementary Material

Figure S1 Western blot results of p65, p-p65, ikB, p-ikB and GAPDH.

Figure S2 Western blot results of JAK2, p-JAK2, STAT3, p-STAT3 and GAPDH.
