# Supplementary figures and images for: Protective Effect of Stachydrine Against Cerebral Ischemia-Reperfusion Injury by Reducing Inflammation and Apoptosis Through P65 and JAK2/STAT3 Signaling Pathway
Source: Front Pharmacol. 2020 Feb 18;11:64. doi: 10.3389/fphar.2020.00064 (PMC7041339; doi:10.3389/fphar.2020.00064)

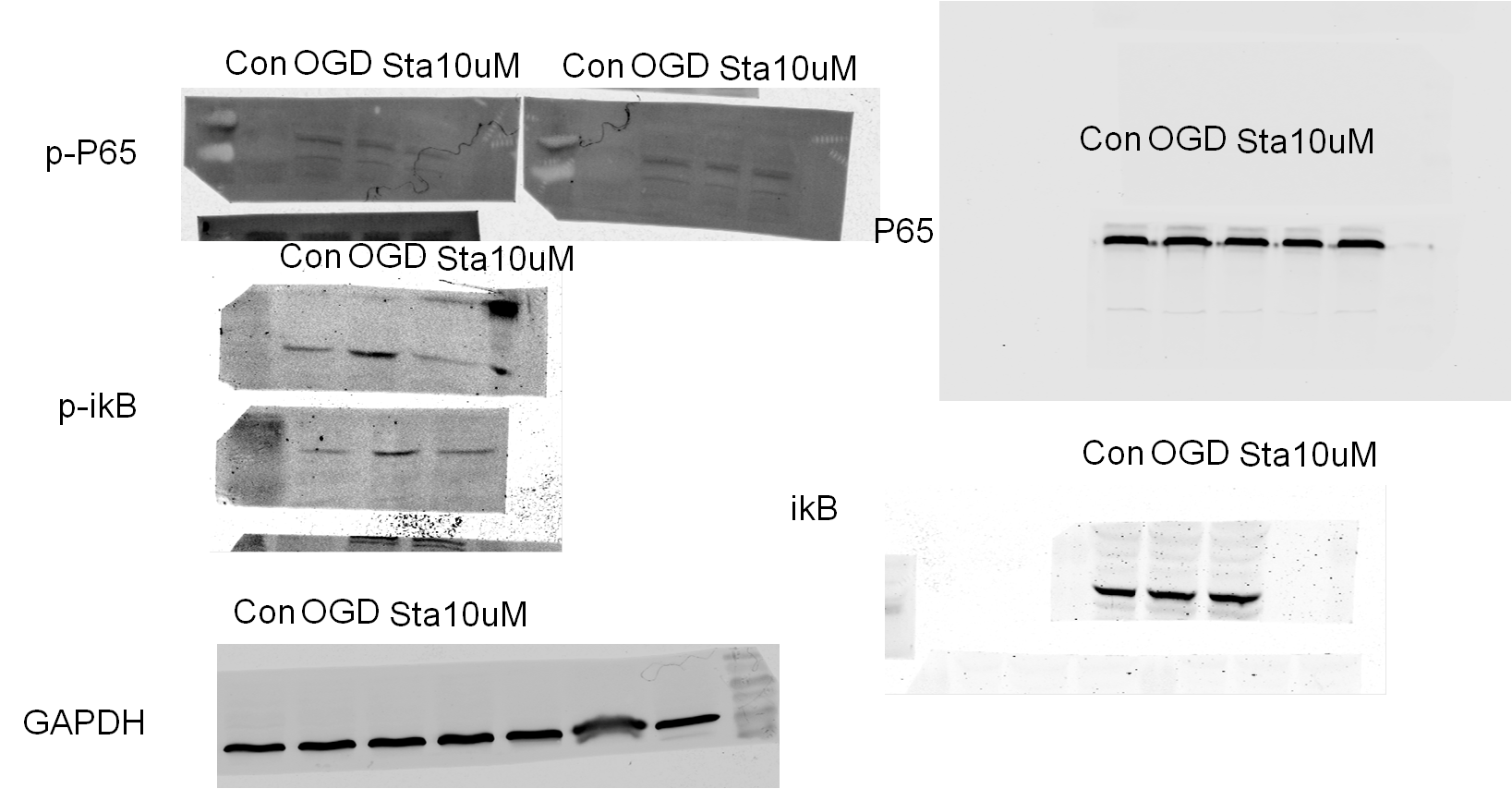

Supplement: Figure S1 — Western blot results of p65, p-p65, ikB, p-ikB and GAPDH. [file Image_1.tif]

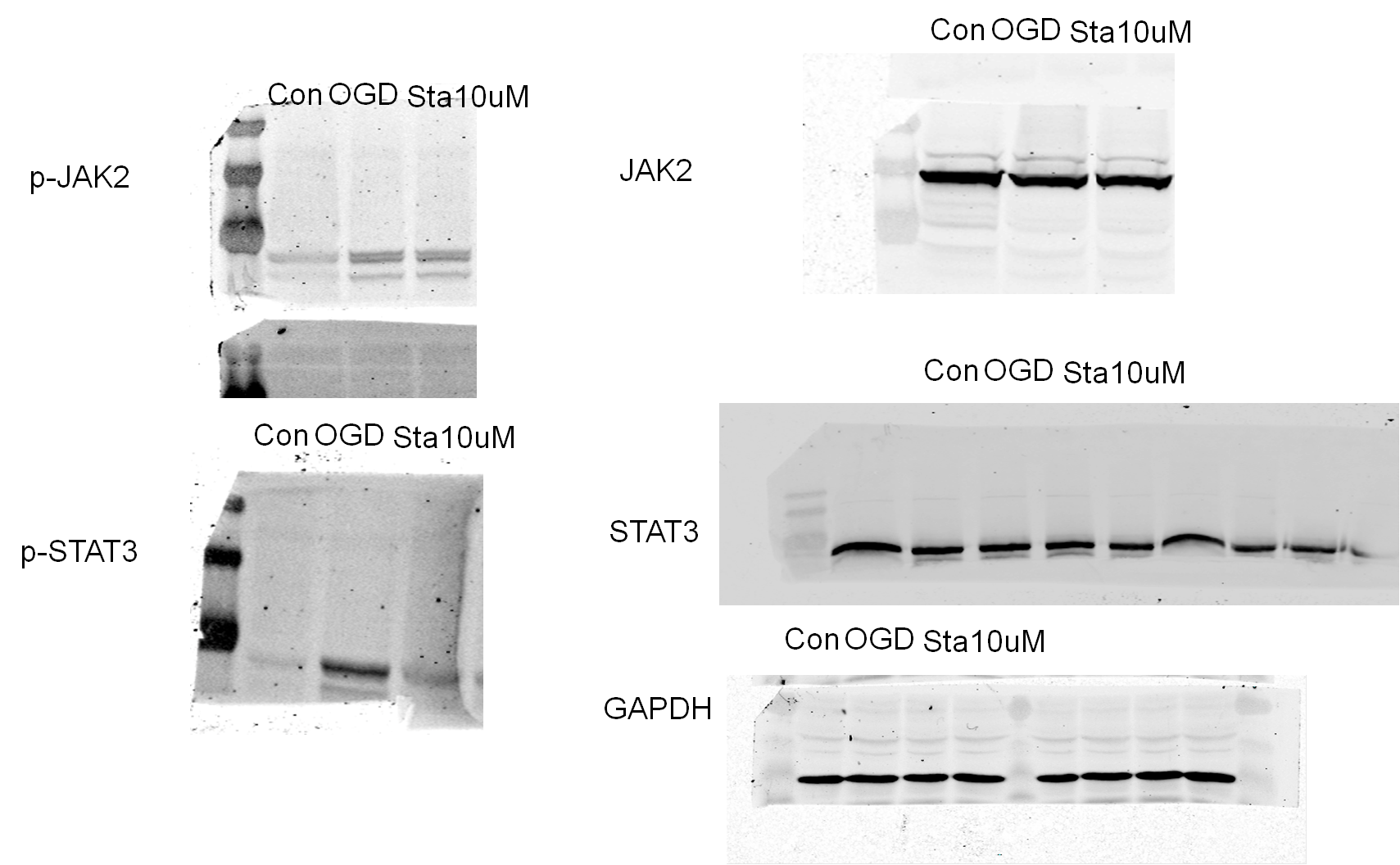

Supplement: Figure S2 — Western blot results of JAK2, p-JAK2, STAT3, p-STAT3 and GAPDH. [file Image_2.tif]
